# Experimental Study on Temperature Effects on NiTi Shape Memory Alloys under Fatigue Loading

**DOI:** 10.3390/ma13030573

**Published:** 2020-01-25

**Authors:** Caikui Lin, Zeqiang Wang, Xin Yang, Haijun Zhou

**Affiliations:** 1 School of Civil Engineering, Wuhan University, Wuhan 430072, China; lincaikui@polychangda.com.cn; 2 Department of Civil Engineering, University of Bristol, Bristol BS1 5QD, UK; lf19903@bristol.ac.uk; 3 Guangdong Provincial Key Laboratory of Durability for Marine Civil Engineering, Shenzhen University, Shenzhen 518060, China; yangxin9210@gmail.com; 4 Institute of Urban Smart Transportation and Safety Maintenance, Shenzhen University, Shenzhen 518060, China

**Keywords:** SMA, fatigue, temperature, strain amplitude, energy consumption

## Abstract

NiTi Shape Memory Alloy (SMA) has been widely studied in the field of structural vibration control, and the results show that the fatigue life of the SMA is a key factor of the vibration control system. In this paper, the fatigue test is carried out in Dynamic Mechanical Analyzer (DMA + 1000) to analyze how the changes of temperature and strain amplitude affecting the main fatigue parameters. The test results show that when the test temperature is higher than Austenite finish temperature (*A_f_*), the fatigue properties of SMAs are significantly affected by temperature. With the increase of temperature, the fatigue life becomes shorter and the energy consumption decreases, while the area of hysteresis curve, the stress amplitude, and effective modulus increase.

## 1. Introduction

Smart materials have received more and more attention in engineering as they can make a useful, reliable, and reproducible response when the materials are stimulated [1,2,3]. The strength of engineering materials is getting higher and higher and the size of the materials can be made thinner and finer, which might cause structural vibration. To reduce structural vibration, the installation of a damper on the structure is a very effective way. However, dampers are often subjected to the continuous action of reciprocating loads in practical applications, causing problems of service life and reliability. Therefore, smart materials are prevalently applied to improve the reliability and durability of the dampers.

Shape Memory Alloy (SMA) is one kind of smart materials. In 1963, Buehler and his colleagues in the US Naval Ordnance Laboratory first discovered that NiTi Alloy with equal atomic ratio has Shape Memory Effect (SME), and named this material Nitinol [4]. After that, more researches attempted to find the properties of SMA. Two most prominent properties of SMA are shape memory effect and pseudoelasticity [5]. Shape memory effect refers to the ability of SMA to restore its original shape by proper heating after deformation. Pseudoelasticity is the ability of SMA to completely restore deformation after large deformation. The reason for these phenomena is the reversible phase transformation between two phases of shape memory alloys, namely, the mutual transformation between the martensite phase and the austenite phase [6,7].

The research and application of shape memory alloy have been extended to the field of civil engineering, with the improvement of production technology and the reduction of cost in the past three decades [8,9]. The application of SMA equipment in passive, active, and semi-active control of engineering structures has been widely reported [10,11]. Pseudoelastic SMA shows strong hysteretic characteristics in loading–unloading cycles and can dissipate a large amount of energy during these cycles. Most of the practical applications of SMA materials in civil engineering belong to passive control [12,13,14].

When SMA is used in a damper for civil engineering structures, its service life and durability become the major focus of engineering society. SMA components are often subjected to continuous cyclic loads in engineering applications, which means the fatigue problem of SMA must be considered to ensure the safety and durability of the structure in practical applications [15]. Most of the reported researches are achieved by controlling stress amplitude [16,17] or strain amplitude [18,19,20] in the set working temperature, then the materials are cyclically loaded to failure [21,22]. As we all know, the working environment is harsh for engineering structures, as the structures are exposed to the variation of environmental temperature; the effects of temperature on SMA fatigue performance cannot be ignored.

As a temperature sensitive material, SMA exhibits different fatigue properties at different temperatures. Miyazaki et al. [23] successfully analyzed the different trends of strain-life of SMA at various test temperatures and indicated that the fatigue life of superelastic SMA specimens increases with the decrease of test temperatures. Casassoi and Marzi [24] came to a similar conclusion. Tobushi et al. [25] derived the equation to quantify the relationship between the fatigue life and strain range, temperature as well as frequency. Kim [26] found the convergence of the strain-life curve after about 10^5^ cycles, which means that the strain–life curve is insensitive to temperature after the numbers of cycles over one hundred thousand. SMA, as a damper material, is supposed to be used at various temperatures. Although some researchers have measured the effect of temperatures on the fatigue life, few people have focused on other fatigue parameters before. This paper investigated the effects of temperature and strain amplitude on the uniaxial tensile fatigue properties of pseudoelastic NiTi shape memory alloys.

## 2. Test Materials

**Figure 1:**
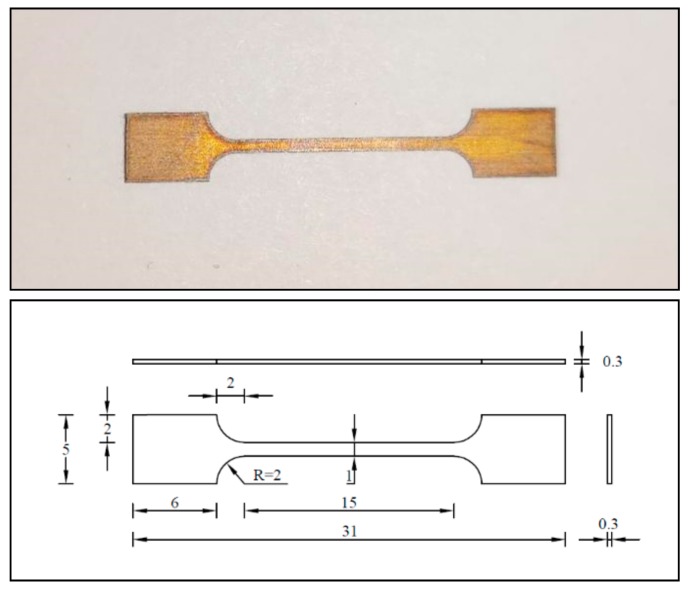
SMA dog bone sheet specimen and dimensions (Unit: mm).

The NiTi shape memory alloy used in the test was manufactured by Beijing Jiyi Technology and Trade Co., Ltd (Beijing, China). It is a nearly equal nickel and titanium atom containing alloy with slightly higher nickel content. Its chemical composition is shown in Table 1. The atomic mass ratio of the nickel element of NiTi shape memory alloy is ~55.980%. Figure 1 shows the customized shape memory alloy dog bone sheet specimens from the manufacturer. The test section of the specimen is 15 mm in length, 1 mm in width and 0.3 mm in thickness.

The transformation temperatures of the SMA specimen were measured by using DSC Q200 thermal analysis system. The test results show that the Martensite start temperature *M_s_* of the test sample is 25.62 °C, the Martensite finish temperature *M_f_* is 6.90 °C, the Austenite start temperature *A_s_* is 19.33 °C, and the Austenite finish temperature *A_f_* is 32.60 °C.

## 3. Test Methods

**Figure 2:**
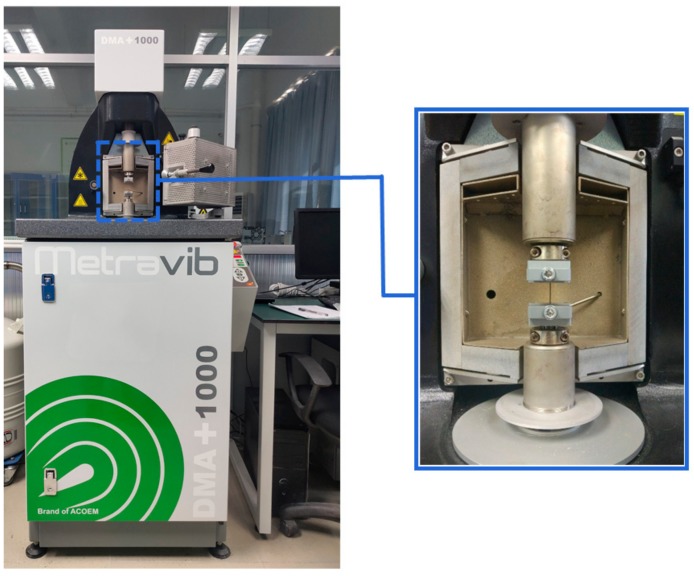
DMA + 1000 and test chamber.

The fatigue test was carried out by Dynamic Mechanical Analyser (DMA + 1000) in Shenzhen University (Figure 2). This instrument can measure the stress response under alternating strain and a series of fatigue parameters such as stress, strain and effective modulus in material fatigue test. The maximum load that DMA + 1000 can apply is 200 N with precision of 0.0001 N, the chamber temperature error does not exceed ±0.1 °C. The advantage of DMA + 1000 is that it can intuitively measure the energy consumption (the loss modulus) during the loading test.

Table 2 lists the tested cases: The tests were a tensile fatigue test controlled by the strain amplitude, a total of nine strain ranges were set, which were 0.4%, 0.5%, 0.6%, 0.7%, 0.8%, 1.0%, 1.2%, 1.6%, and 2.0%, respectively. The test temperatures were set as *T* = 35 °C, 40 °C, 45 °C, 50 °C; Δ*T* = 2.4 °C, 7.4 °C, 12.4 °C, and 17.4 °C, respectively, where Δ*T = T − A_f_* is the temperature difference between the test and the Austinite finish temperature. The above four temperatures are higher than the SMA austenite finish temperature *A_f_*, i.e., Δ*T > 0*, which ensured that the SMA is in a completely austenitic state during the test. All tested cases were loaded with a sine wave at a loading frequency of 5 Hz. Effects of temperature, strain amplitude on fatigue life, stress amplitude, stiffness, energy dissipation, and other fatigue parameters of SMA were derived.

To ensure that SMA specimens have stable pseudoelasticity in fatigue test [27] and avoid compression caused by residual deformation during the test, the mechanical training of the SMA specimens must be performed before tests. The training process of all the test samples was carried out in a constant temperature test chamber at 40 °C. Mechanical training was carried out by tension cyclic loading. The loading and unloading rates were both 0.2 mm/s and the maximum tensile strain was 4%. A total of 30 cycles were carried out to make the SMA specimens have smaller residual strain and stable hysteretic curve at the end of training. After the mechanical cycle training of SMA, the fatigue tests were carried out immediately according to the working conditions listed in Table 2.

## 4. Test Results

### 4.1. Fatigue Life

**Figure 3:**
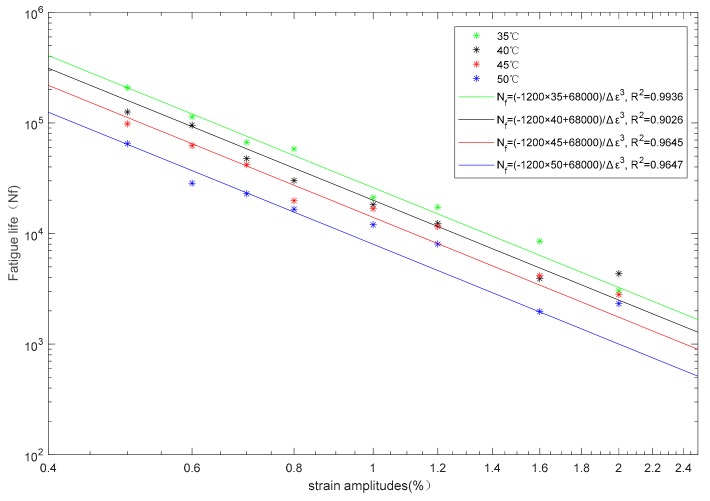
Fatigue life curves at different temperatures for various strain amplitudes.

The fatigue life test results for various temperatures and strain amplitudes are listed in Table 3. At four different temperatures, the scatter plots of strain amplitude and fatigue life are obtained in Figure 3 with log-log coordinates. The fatigue life is calculated as follows by regression of the experimental results.

(1) $$N_{f}=\left(-1200\times T+68,000\right)\times \Delta \varepsilon^{-3},$$

where $N_{f}$ is fatigue life, T is temperature (°C), and Δε is strain amplitude. The coefficient of determination $R^{2}>0.9$, indicated in Equation (1), could well predict the fatigue life of tested SMA specimen. When the strain amplitude is 0.4%, the fatigue life of SMA specimens at 35 °C, 40 °C and 45 °C are larger than 2 million cycles. Because the specific cycles of fatigue life are unknown, we do neglect these data in the regression Equation (1).

Figure 3 shows that both temperature and strain amplitude have significant effects on the fatigue life of pseudoelastic SMA. To be more specific, as strain amplitudes increase from 0.4% to 0.6%, the fatigue life of SMA decreases rapidly by an order of magnitude. As the strain amplitude continues increasing, the fatigue life continuing decreases to several thousand for strain amplitude of 2.0%. It also can be seen intuitively that the fatigue life of the SMA specimen has strong temperature sensitivity: within the test temperature range (35–50 °C), as the temperature increases, the fatigue life of SMA decreases. Furthermore, the smaller the strain amplitude, the more obvious this trend is, and the larger the effect of temperature on fatigue life.

### 4.2. Hysteresis Curves

**Figure 4:**
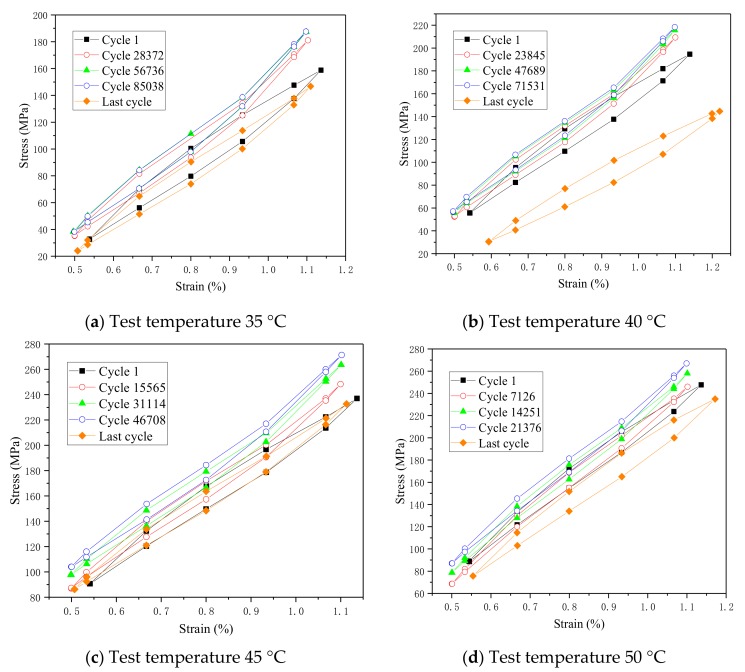
Hysteretic curves of different test temperatures: (**a**) 35 °C, (**b**) 40 °C, (**c**) 45 °C, and (**d**) 50 °C.

**Figure 5:**
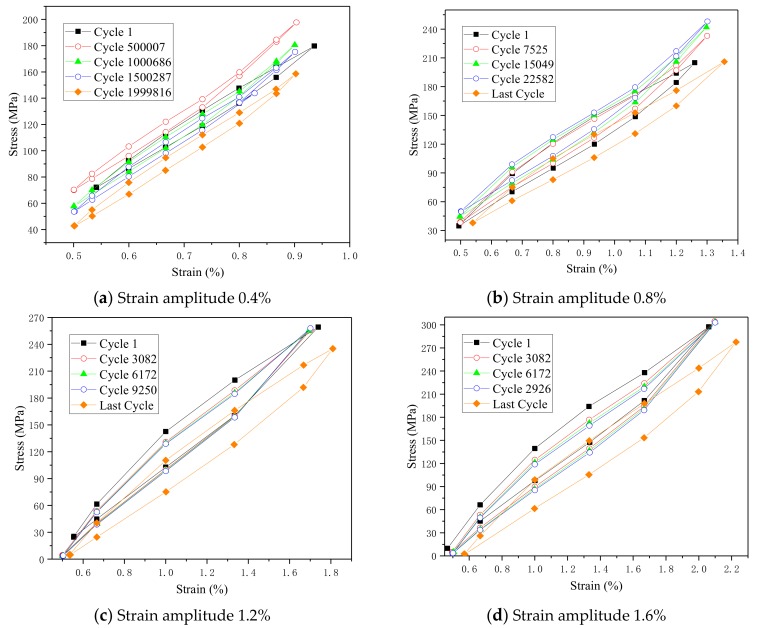
Hysteretic curves of different strain amplitudes: (**a**) 0.4%, (**b**) 0.8%, (**c**) 1.2%, and (**d**) 1.6%.

The hysteresis curves in Figure 4 are obtained at a strain amplitude of 0.6% with four different temperatures, whereas Figure 5 shows hysteretic curves of different strain amplitudes at 40 °C. Both these figures confirm to the typical shape of the hysteresis curve for austenitic SMA pseudoelastic behavior. Figure 4 shows five cycles of each stress–strain curves of the fatigue life: the first cycle, followed by the quarter, half, 3 quarter, and the last cycle before failure of the fatigue life, respectively. As the loading cycles increases, it can be seen that the hysteresis loop area is gradually reduced and the width is narrowed for the first few cycles. When the specimen is close to failure, the maximum stress of the hysteretic loops decreases, while the width of the hysteretic loops increases and the area of the hysteretic loop increases. Comparing the differences of hysteretic loops at four test temperature in Figure 4, we found that the area of hysteretic loops significantly decreased from 35 °C to 50 °C in the relatively identical period of fatigue life. In addition, Figure 5 shows that the area of hysteretic loops remarkably increases in the relative same loading cycle with the strain amplitude raising from 0.4% to 1.6%.

### 4.3. Stress Amplitude

**Figure 6:**
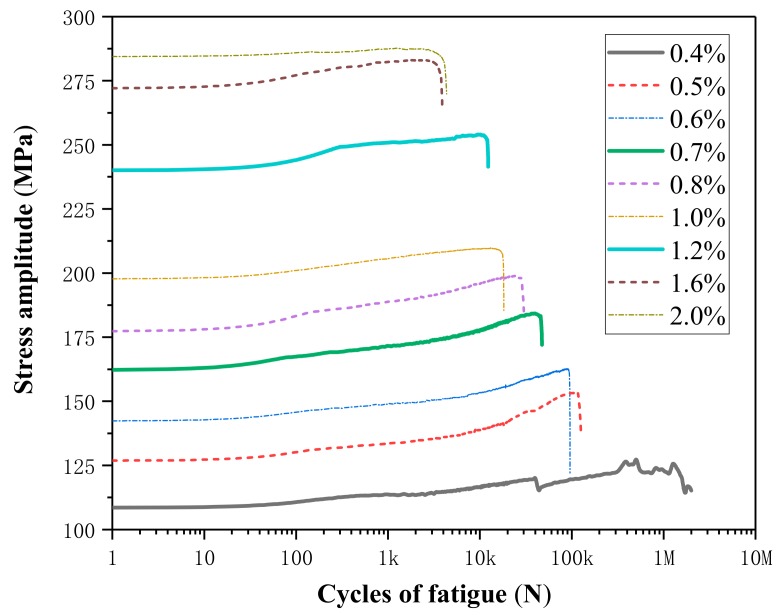
Stress amplitude curves of different strain amplitudes at 40 °C.

The stress amplitude is defined as the maximum stress minus the minimum stress in one loading cycle. When the temperature is constant, the relationship between the stress amplitude and the number of cycles is shown in Figure 6. It clearly shows that the stress amplitude increases slowly with the increase of the number of cycles. This means that the deformation resistant ability of SMA increases continuously during the cyclic loading process, and could provide enough stress response to restore deformation even after many loading cycles in engineering. The increasing of stress amplitudes varies from 1.9% to 18.9%. To be more specific, the proportion of stress amplitude increase is ~18.9% when the strain amplitude is 0.4%; as the strain amplitude rises to 2.0%, the proportion of stress amplitude increment declines to 1.9%. This means that the cyclic hardening phenomenon is more obvious at lower strain amplitude. Furthermore, we found that the larger the strain amplitude, the larger the stress amplitude.

**Figure 7:**
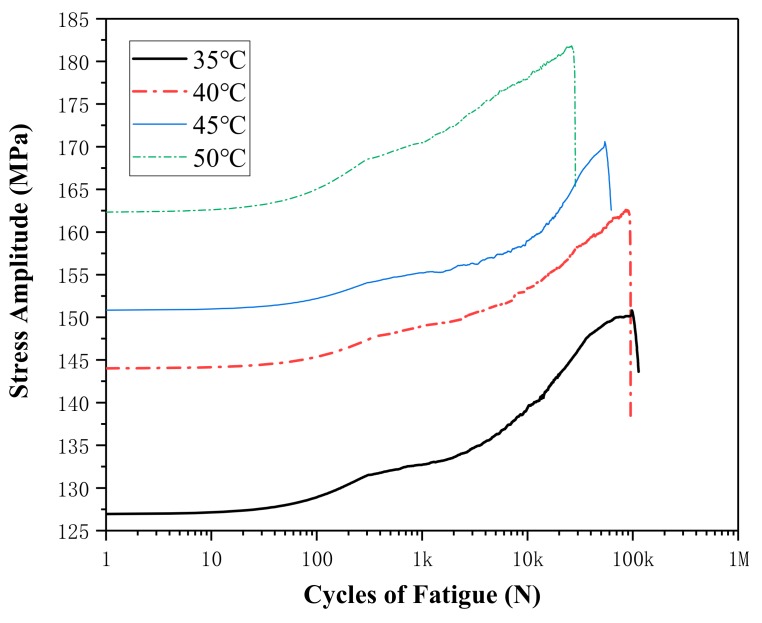
Stress amplitude curves at different temperatures for strain amplitude of 0.6%.

Figure 7 shows the effects of temperature on stress amplitude at strain amplitudes of 0.6%. The stress amplitude increases with the increasing of the test temperature, from about 126.9 MPa at 35 °C, 144.0 MPa at 40 °C, 150.8 MPa at 45 °C to 162.3 MPa at 50 °C for the first cycle loading. The stress amplitude increases slowly as cycle number increases. When the cycle number is 10 k, the stress amplitude is 139.4 MPa, 153.4 MPa, 159.0 MPa, and 177.9 MPa.

### 4.4. Effective Modulus

The effective modulus reflects the ability of a material or structure to resist elastic deformation under stress. The effective modulus *E* of the specimen in the fatigue test is calculated in this test

**Figure 8:**
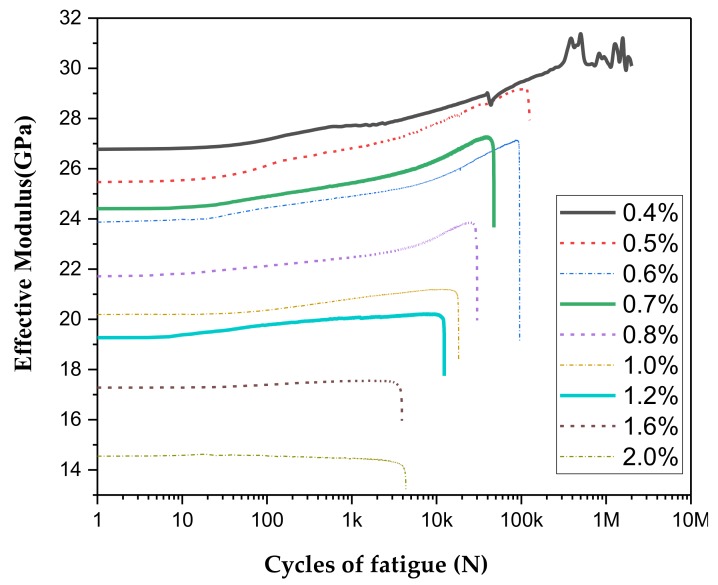
Effective modulus curves of different strain amplitudes at 40 °C.

(2) E=Δσ/Δε

in which $\Delta \sigma =\frac{F_{\max}-F_{\min}}{S}$, $\Delta \varepsilon =\frac{l_{\max}-l_{\min}}{l}$. *F*_max_ is the maximum value of a force in a cycle, *F*_min_ is the minimum. *l*_max_ is the maximum value of the displacement in a cycle, *l*_min_ is the minimum, **S** is the cross-sectional area of the specimen, and *l* is the effective length of the specimen. The relationship between effective modulus and cycle number is shown in Figure 8. Equation (2) shows that the effective modulus is proportional to the stress amplitude, so the curve of the effective modulus and the curve of the stress amplitude have the same trend. It can be seen that the effective modulus of SMA specimens keeps a certain level during the test process. The effective modulus increases slowly with the increase of the test cycle, which means the ability to resist deformation gradually increases in this process. The effective modulus finally plunges rapidly when approaching to the failure. As can be seen from the previous section, the stress amplitude and strain amplitude are proportional, but the effective modulus is different from the strain amplitude. The effective modulus is inversely proportional to the strain amplitude. The larger the strain amplitude, the smaller the effective modulus. This is mainly due to the particularity of the SMA material. Stress induces a phase change and generates a phase change platform.

However, we can also see the effective modulus curve 0.6% and 0.7% have not followed the trends, we do think this was also due the randomness. Another important fact may be due to the fact that the stress-induced Austenite to Martensite is happened around the strain of 1.0%, which was quite close to the maximum strain of the two loaded cases. We think this may increase the sensitivity of SMA to randomness.

### 4.5. Energy Dissipation

Pseudoelastic SMA is a good material for dissipating energy during structural vibration. Therefore, in the design of SMA components, energy consumption is a crucial parameter. The loss modulus describes the phenomenon that the energy loss is transformed into heat when the material is deformed. The calculation formula is shown in Equation (3):

(3) $$E^{\prime \prime }=\left|E\right|\times \sin\delta ,$$

where $E^{\prime \prime }$ is loss modulus, E is Young’s modulus, and δ is phase angle (phase difference between the dynamic stress and the dynamic strain in a viscoelastic material subjected to a sinusoidal oscillation). Generally, the loss modulus value is proportional to the area enclosed by the hysteresis curve (stress–strain curves in Figure 4 and Figure 5).

**Figure 9:**
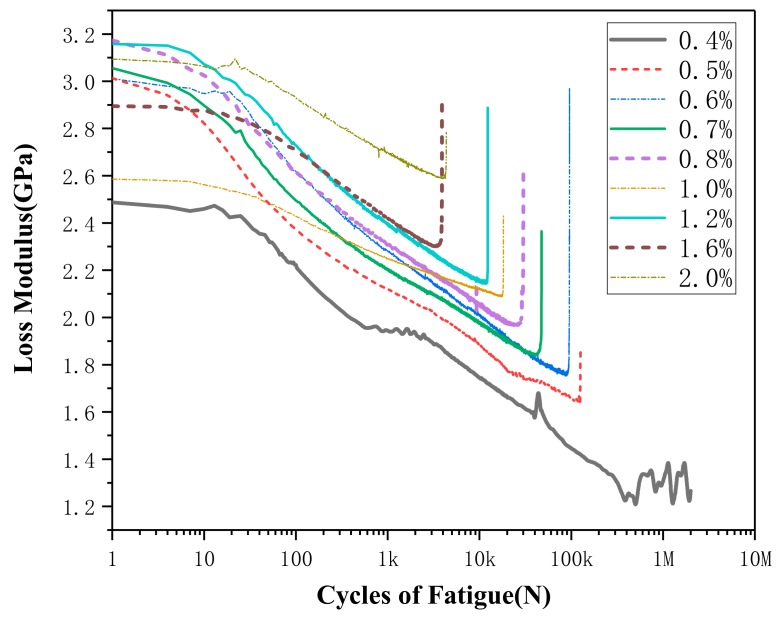
Loss modulus curve of different strain amplitudes at 40 °C.

**Figure 10:**
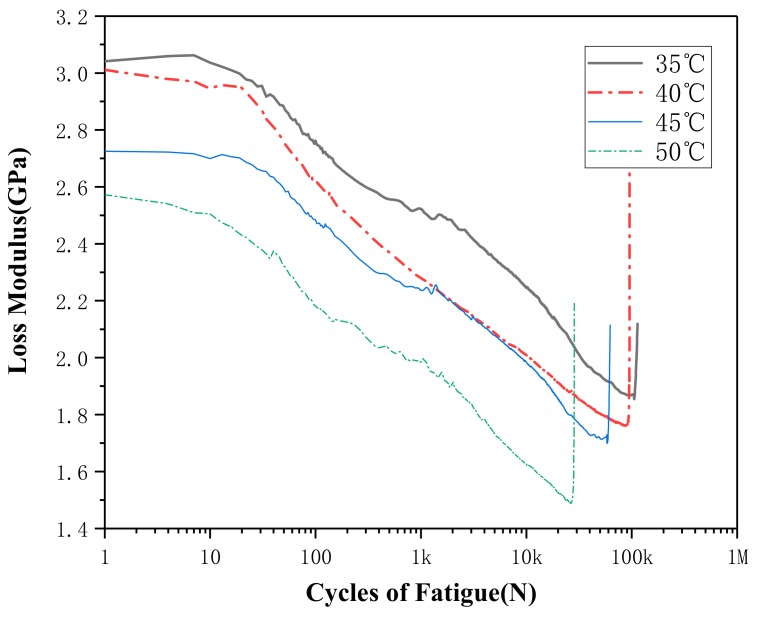
Loss modulus curves at different temperatures for strain amplitude of 0.6%.

The loss modulus curves are shown in Figure 9 and Figure 10. As can be seen from figures, the loss modulus curves under all working conditions are similar. The loss modulus gradually decreases as loading cycle number increases, and then suddenly it rises sharply before it is about to be destroyed. Figure 8 reveals that the loss modulus has a gradual rise trend when the strain amplitude increases. On the contrary, it can be seen from Figure 10 that the loss modulus decreases when the temperature increases. Figure 8 show the loss modulus curve of 1.0% curve has not followed the trends, the reason may be due to fact that SMA is a sensitive smart material; it shows some kinds of uncertainty [28,29]. The uncertainty may come from the SMA material itself or during the test process: the production and heat treatment process, the training process of SMA before the test, test temperature, the initial stress during samples installation, etc.

## 5. SEM Analysis

**Figure 11:**
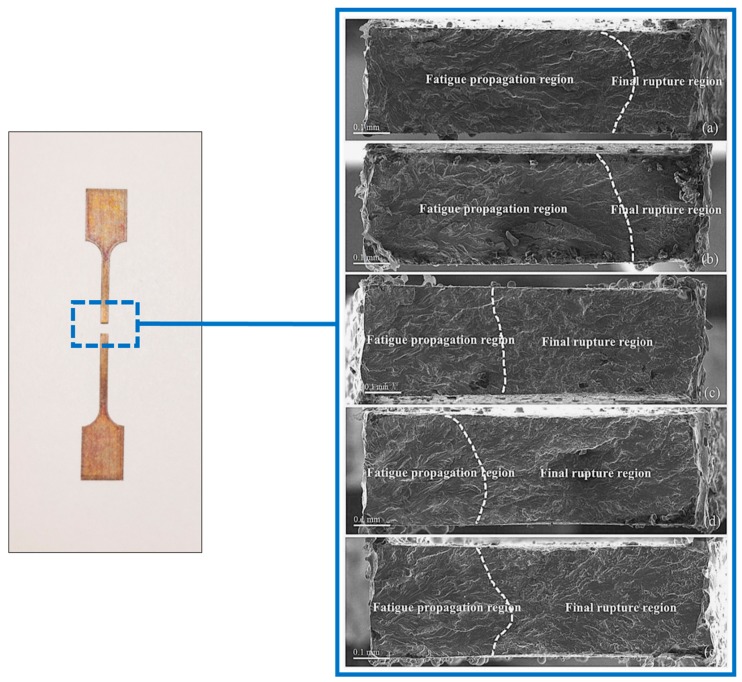
SEM micrograph of fracture of different strain amplitude: (**a**) 0.6%, (**b**) 0.8%, (**c**) 1.2%, (**d**) 1.6%, and (**e**) 2.0%.

The left side of Figure 11 shows the specimen after failure. It can be seen that there is no necking deformation in the failure section of the specimen, and the failure is brittle. Figure 11 shows SEM micrograph of the fracture surface obtained by testing the samples at different strain amplitudes (0.6%, 0.8%, 1.2%, 1.6%, and 2.0%). From the macroscopic point of view, there is no significant change in the area of the fatigue fracture of the SMA specimen. The fracture does not produce plastic deformation, which is consistent with the characteristics of metal fatigue failure. Figure 11 also clearly shows that there are three regions with completely different characteristics for the fatigue fracture of the SMA specimen, namely, the fatigue source zone, fatigue propagation zone, and fatigue fracture zone, which represent three different processes of fatigue failure over time. Due to the surface defects generated during the manufacturing process, the cracks of the specimen are initiated from the surface and form a fatigue source zone at the edge of the section. This zone is a relatively flat small area, which appears on the left side of the figure. After that, the fatigue cracks continue to grow along crystallographic planes during the fatigue process, a fatigue propagation zone with fatigued ridges is formed. As the fatigue crack further propagates, the residual effective area of SMA decreases and the loading stress increases. Once the loading stress exceeds the ultimate stress of SMA, the specimen is instantaneously destroyed, forming a final rupture region with a typical dimple structure on the right side of the figure.

When the strain amplitude is 0.6%, the area of the fatigue source zone and the fatigue propagation zone exceeds half of the total cross-sectional area, which is about three-quarters of the total cross-sectional area. However, as the strain amplitude level increases, the specimen fatigue source area and the area of the fatigue expansion accounts for a smaller proportion of the total area. When the strain amplitude is 2.0%, the proportion of these two zones in the total area is only about a quarter. It can also be observed that the fringe depth of the fatigue propagation zone has a certain relationship with the strain amplitude. As the strain amplitude increases, the depth of the fatigue fringes tends to become shallower.

**Figure 12:**
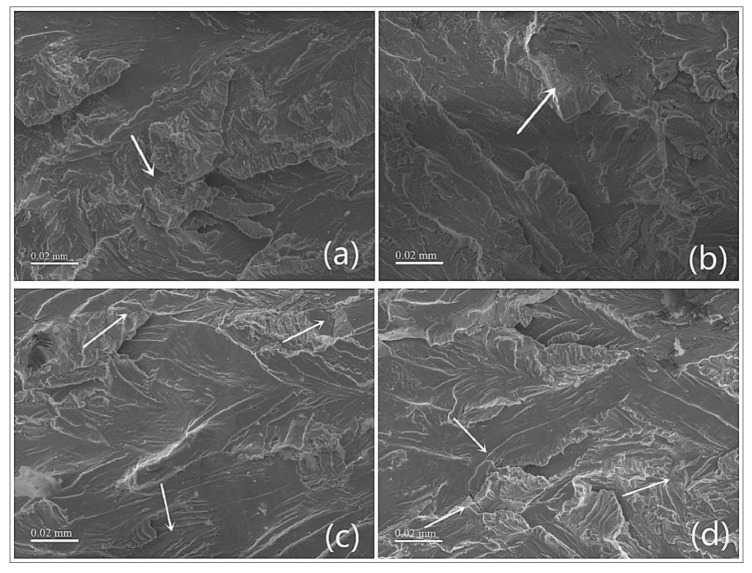
Crack distributions in fatigue propagation zone at different temperatures: (**a**) 35 °C, (**b**) 40 °C, (**c**) 45 °C, and (**d**) 50 °C.

The effect of temperature on fatigue performance may be related to changes in microstructure, such as crystal diffusion, aging, dislocation reconstruction, recrystallization, and other factors, which further cause the microstructure transformation of the material [21]. The instability of the microstructure accelerates the destruction of the structure. From Figure 12, the micromorphology of the fatigue propagation zone at different temperatures can be seen when the strain amplitude is 0.6%. By comparing the fatigue fractures under the four temperature conditions, we found that there are more cracks in the fatigue propagation zone at higher temperature. The cracks are longer and deeper. This is mainly because the increase in temperature makes the austenite more stable and increases the stress associated with the austenite–martensite (A–M) interface, accelerating the fatigue nucleation in the A–M region [30]. Therefore, the high temperature accelerates the crack initiation and development to the inside of the SMA specimen, which results in a reduction in the fatigue life of the sample at higher temperatures.

## 6. Conclusions

The uniaxial tensile fatigue tests of SMAs were carried out with different temperatures and different strain amplitudes, the following conclusions were drawn.

1. The fatigue life of SMA is sensitive to temperature and strain amplitude. When the temperature or strain amplitude increases, the fatigue life is obviously decreased.
2. Both the stress amplitude and the effective modulus show a gradual increase during the fatigue test. Moreover, the stress amplitude and effective modulus are proportional to the temperature. The higher the temperature, the higher the stress amplitude and the effective modulus.
3. Although the loss modulus generally shows a decreasing trend, it maintains a certain level (~60% of the initial value) in the middle and later stages. This indicates that SMA has stable vibration damping performance in whole working life. The temperature also has a remarkable effect on the loss modulus: with the increase of temperature, the loss modulus declines.
4. The fracture of SMA conforms to the characteristics of metal fracture. The high temperature accelerates the initiation and expansion of the crack, which results in the shorter fatigue life of the SMA.

Although we find that the SMA has prominent fatigue properties as one kind of damping material based on the above conclusions, SMAs are supposed to be optimized in terms of the fatigue performance for more complex working conditions as the environmental temperature fluctuates during working life. The fatigue performance of SMA under such kind of temperature conditions are still needs further exploration.

## Figures and Tables

**Table 1:** NiTi SMA compositions.

Elements	Ni	C	O	Fe	Co	Cr
Contents (w/%)	55.980	0.042	0.038	<0.010	<0.005	<0.005
Elements	Cu	Nb	N	H	Ti	-
Contents (w/%)	<0.005	<0.005	<0.003	<0.001	Balance	-

**Table 2:** Test cases.

Cases	*T* (°C)	*ε*_min_ (%)	*ε*_max_ (%)	Δε (%)
A1	35	0.5	0.9	0.4
A2		0.5	1.0	0.5
A3		0.5	1.1	0.6
A4		0.5	1.2	0.7
A5		0.5	1.3	0.8
A6		0.5	1.5	1.0
A7		0.5	1.7	1.2
A8		0.5	2.1	1.6
A9		0.5	2.5	2.0
B1	40	0.5	0.9	0.4
B2		0.5	1.0	0.5
B3		0.5	1.1	0.6
B4		0.5	1.2	0.7
B5		0.5	1.3	0.8
B6		0.5	1.5	1.0
B7		0.5	1.7	1.2
B8		0.5	2.1	1.6
B9		0.5	2.5	2.0
C1	45	0.5	0.9	0.4
C2		0.5	1.0	0.5
C3		0.5	1.1	0.6
C4		0.5	1.2	0.7
C5		0.5	1.3	0.8
C6		0.5	1.5	1.0
C7		0.5	1.7	1.2
C8		0.5	2.1	1.6
C9		0.5	2.5	2.0
D1	50	0.5	0.9	0.4
D2		0.5	1.0	0.5
D3		0.5	1.1	0.6
D4		0.5	1.2	0.7
D5		0.5	1.3	0.8
D6		0.5	1.5	1.0
D7		0.5	1.7	1.2
D8		0.5	2.1	1.6
D9		0.5	2.5	2.0

**Table 3:** Fatigue life of SMA specimens.

	Strain Amplitudes	0.4%	0.5%	0.6%	0.7%	0.8%	1.0%	1.2%	1.6%	2.0%
Temperature										
35 °C		>2 × 10^6^	208,411	113,607	66,718	58,198	21,082	17,302	8517	3031
40 °C		>2 × 10^6^	125,602	95,376	47,512	30,101	18,191	12,334	3901	4339
45 °C		>2 × 10^6^	98,145	62,256	41,908	19,787	16,758	11,524	4155	2806
50 °C		126,356	64,904	28,501	22,878	16,586	11,999	8041	1969	2320
